# Differential clusterization of soluble and extracellular vesicle-associated cytokines in myocardial infarction

**DOI:** 10.1038/s41598-020-78004-y

**Published:** 2020-12-03

**Authors:** Anna Lebedeva, Wendy Fitzgerald, Ivan Molodtsov, Alexander Shpektor, Elena Vasilieva, Leonid Margolis

**Affiliations:** 1 Laboratory of Atherothrombosis, Moscow State University of Medicine and Dentistry, 11/6 Yauzskaya Street, Moscow, Russia 119027; 2 Section On Intercellular Interactions, Eunice Kennedy Shriver National Institute of Child Health and Human Development, National Institutes of Health, Bethesda, MD 20892 USA; 3 N.F. Gamaleya Federal National Research Centre for Epidemiology and Microbiology, 18 Gamaleya Street, Moscow, Russia 123098; 4 Present Address: Department of Internal Medicine and Cardiology, Charité University of Medicine Berlin, Augustenburger Platz 1, 13353 Berlin, Germany

**Keywords:** Myocardial infarction, Chemokines, Cytokines, Extracellular signalling molecules

## Abstract

A proinflammatory dysregulation of cytokine release is associated with various diseases, in particular with those of infectious etiology, as well as with cardiovascular diseases (CVD). We showed earlier that cytokines are released in two forms, soluble and in association with extracellular vesicles (EVs). Here, we investigated the patterns of expression and clustering of soluble and EV-associated cytokines in patients with ST-elevation myocardial infarction (STEMI). We collected plasma samples from 48 volunteers without CVD and 62 patients with STEMI, separated soluble and EV fractions, and analyzed them for 33 cytokines using a multiplexed bead-based assay. We identified soluble and EV-associated cytokines that are upregulated in STEMI and form correlative clusters. Several clustered soluble cytokines were expressed almost exclusively in patients with STEMI. EV-associated cytokines were largely not affected by STEMI, except for pro-inflammatory cytokines IL-6, IL-18, and MIG, as well as anti-inflammatory IL-2 that were upregulated in a correlated fashion. Our results demonstrated that soluble cytokines in patients with STEMI are upregulated in a coordinated fashion in contrast to the mainly unaffected system of EV-associated cytokines. Identification of cytokine clusters affected differently by STEMI now permits investigation of their differential contributions to this pathology.

## Introduction

Immune activation associated with the release of cytokines is a hallmark of many pathologies^1–6^. In extreme cases, this release becomes highly dysregulated worsening the clinical course of the disease^7^. In other pathologies such abnormal cytokine release, although not that dramatic, is also associated with the disease progression. In particular, the development and progression of human atherosclerotic plaques, the principal cause of cardiovascular diseases (CVD), has been shown to be strongly associated with the inflammatory process and the release of various pro-inflammatory cytokines (predominantly chemokines) that, among other functions, facilitate migration of immune cells inside the plaque and contribute to plaque destabilization^8,9^. Upregulation of these cytokines has been shown to be associated with the risk of development of CVD^10^ and of one of its major complications, acute coronary syndrome (ACS)^11,12^.

Recently, it was demonstrated that cytokines are released not only in soluble form but also are associated with extracellular vesicles (EVs). These vesicles transfer encapsulated molecules, in particular cytokines as well as RNA, from one cell to another^13–18^. Cytokine packaging in EVs is a regulated process, and the profile of cytokines in EVs is different from that of soluble molecules^18,19^.

Moreover, we and others have found that the numbers of EVs of diverse origin are significantly increased in patients with different forms of CVD, especially in ACS^20–24^, and this increase is associated with the risk of development of CVD complications^25,26^. Changes in EV-associated cytokines have been reported for several pathologies including infectious diseases^17,18,27^. Moreover, complex interconnections between groups of soluble and EV-associated cytokines have been found previously for several diseases^19,28^, but the content of EVs has not been systematically studied for CVD. Here, we studied the patterns of cytokines released as soluble molecules and in association with EVs in patients with the most severe form of CVD, the ST-elevation myocardial infarction (STEMI). We determined the differences in the levels of various cytokines and analysed their clusters of co-expression separately in EV-associated and soluble forms. This approach allowed us to report on the distinct patterns of cytokines released as soluble molecules and on cytokine packaging into EVs in patients with STEMI.

## Results

### Evaluation of cytokines in soluble and EV-associated forms in patients with STEMI and controls

We measured 33 cytokines in plasma of 62 patients with STEMI and of 48 controls without CVD. We separated plasma into EV and EV-depleted fractions using ExoQuick. Earlier we demonstrated that ExoQuick did not sediment soluble cytokines, and soluble cytokines did not non-specifically adhere to EVs^19^.

Cytokine concentrations were measured in soluble form and EV-associated form and compared between different groups using a multiplexed bead-based assay. However, among all the evaluated cytokines, the concentrations of IL-4, IL-7, IL-17, ITAC, and MIP-3α were below the lower limit of detection of the assay (LLOD) in more than 90% of all samples in both EV-associated and soluble forms. Those cytokines were therefore excluded from the analysis. The other 28 cytokines were detected in a larger fraction of individuals (see Supplementary Table S1 online) and were further analyzed in soluble and EV-associated forms.

**Figure 1:**
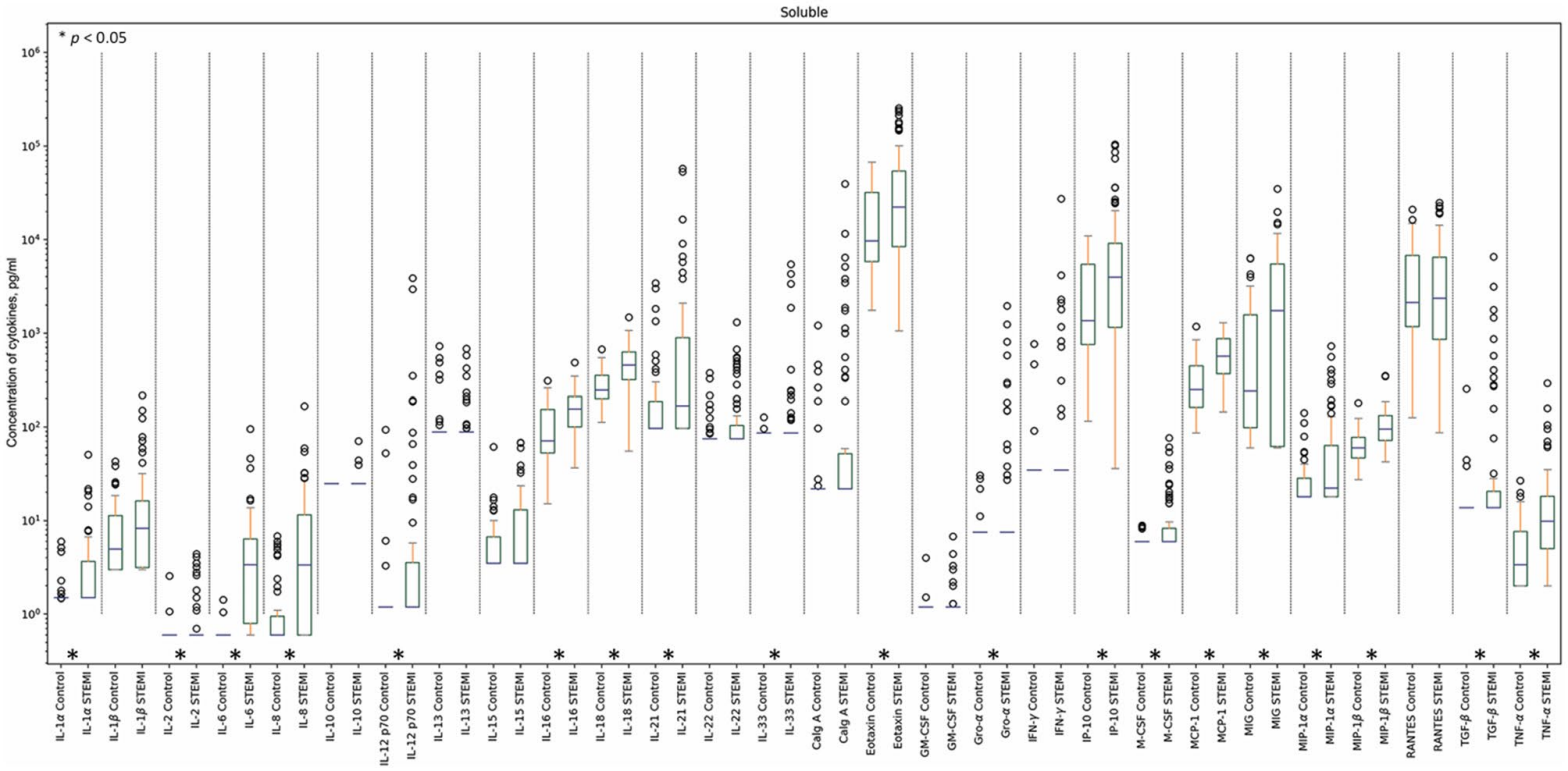
Soluble cytokine concentrations in patients with STEMI and controls. Data are presented as medians and IQR for all the soluble cytokines in the STEMI group and the control group. Asterix indicates *p*-values < 0.05.

**Figure 2:**
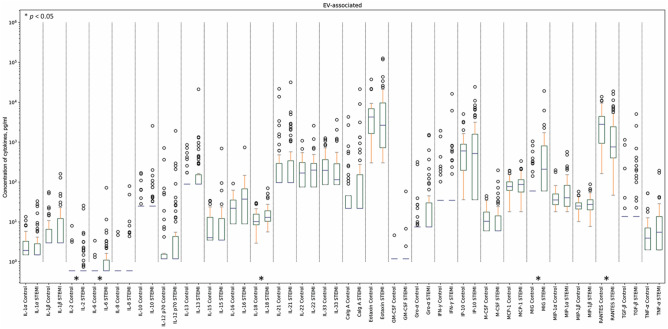
EV-associated cytokine concentrations in patients with STEMI and controls. Data are presented as medians and IQR for all the EV-associated cytokines in the STEMI group and the control group. Asterix indicates *p*-values < 0.05.

**Figure 3:**
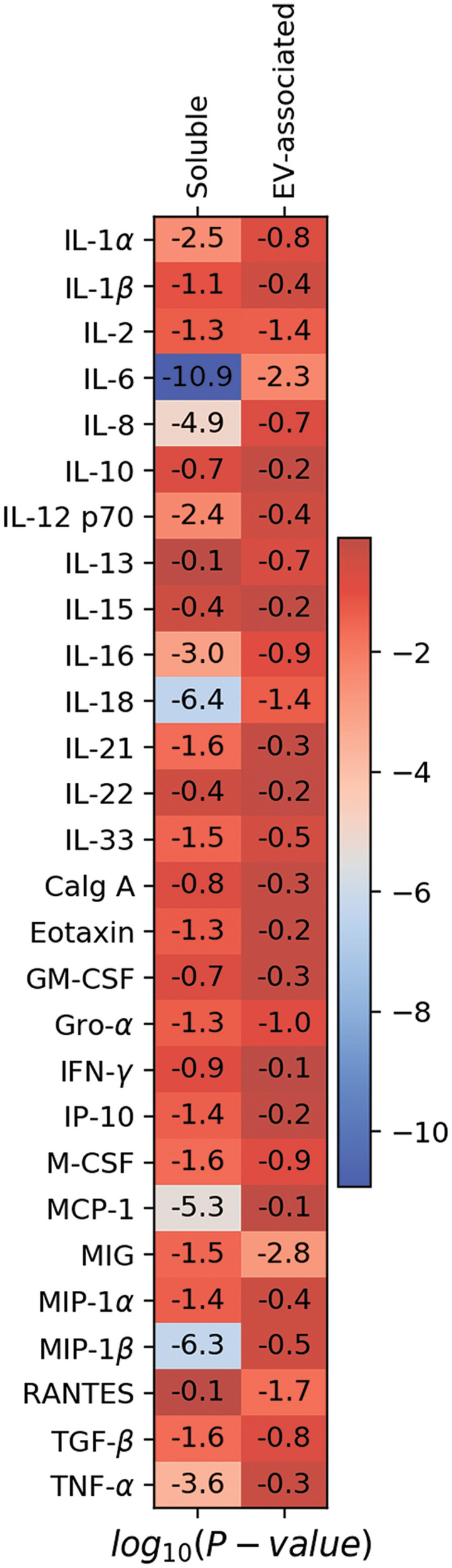
Comparison of cytokine concentrations between patients with STEMI and controls. Shown is a heatmap of *p*-values for the Mann–Whitney U-test for comparisons of the levels of soluble and of EV-associated cytokines between the STEMI group and the control group; *p*-values are presented in log10-scale with Benjamini–Hochberg correction. Log_10_ *p*-values ≤ − 1.3 correspond to *p*-values < 0.05.

First, we performed a comparison of cytokine concentrations between the group of patients with STEMI and the control group. We found that there were significantly higher amounts of IL-1α, IL-2, IL-6, IL-8, IL-12p70, IL-16, IL-18, IL-21, IL-33, Eotaxin, Gro-α, IP-10, M-CSF, MCP-1, MIG, MIP-1α, MIP-1β, TGF-β, and TNF-α in soluble form in plasma of patients with STEMI than in plasma of control volunteers (log_10_ *p* ≤ − 1.3 for the heatmap) (Figs. 1 and 3, also see Supplementary Table S1 online). Four of these cytokines (IL-2, IL-6, IL-18, and MIG) in EV-associated form were also found in significantly higher concentrations in patients with STEMI than in controls. Only RANTES, which was present in similar concentrations in soluble form in both groups of individuals, was lower in EV-associated form in patients than in controls (log_10_ *p* ≤ − 1.3 for the heatmap) (Figs. 2 and 3, also see Supplementary Table S1 online).

**Figure 4:**
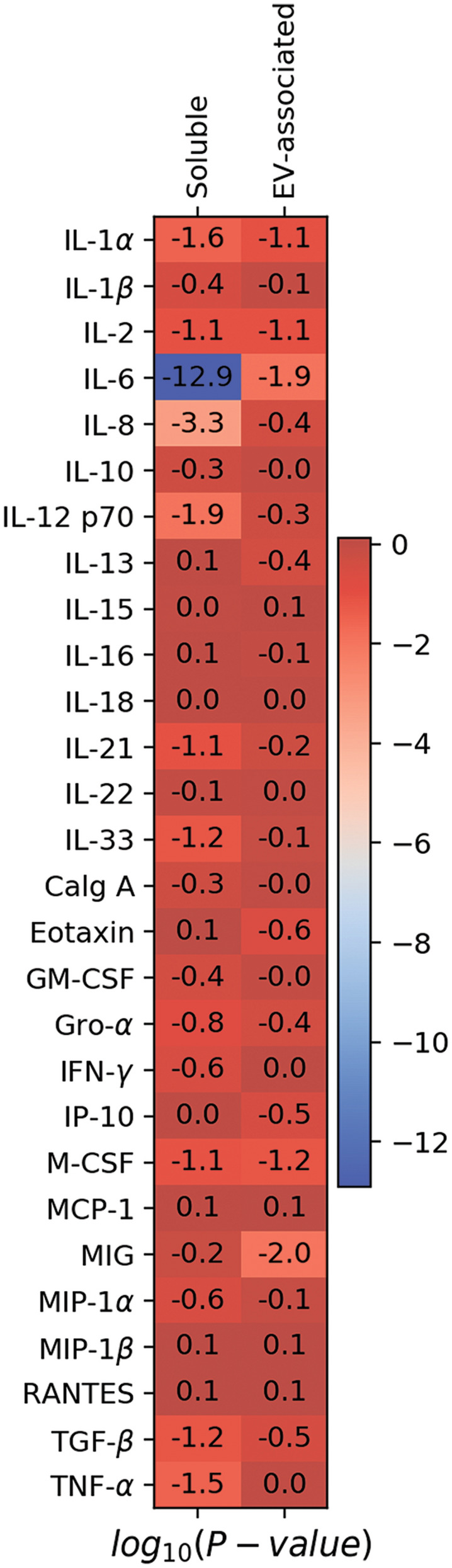
Comparison of frequency of cytokine detection between patients with STEMI and controls. Shown is a heatmap of *p*-values for Fisher exact test of association of the frequencies of cytokine detection in either soluble or EV-associated form between the STEMI group and the control group; *p*-values are presented in log10-scale with Benjamini–Hochberg correction. Log_10_ *p*-values ≤ − 1.3 correspond to *p*-values < 0.05.

For further analysis of all the cytokines, including those that were detected in the minority of the samples, we also compared the frequencies of cytokine detection above the threshold level in patients with STEMI with those in controls. We found that the frequencies of detection of soluble IL-1α, IL-6, IL-8, IL-12p70, and TNF-α were significantly higher in patients with STEMI than in controls (41.9% vs 14.6%, 75.8% vs 4.2%, 71.0% vs 29.2%, 37.1% vs 8.3%, 85.5% vs 60.4%, respectively; *p* < 0.05). However, for all EV-associated cytokines except two (IL-6 and MIG), there was no difference between the numbers of samples from controls and patients with STEMI in which these cytokines were detected. IL-6 and MIG were detected more frequently within the EV fraction of patients with STEMI than in that of controls (32.3% vs 6.2% and 54.8% vs 22.9%, respectively; *p* < 0.05) (Fig. 4, also see Supplemantary Table S1 online).

**Figure 5:**
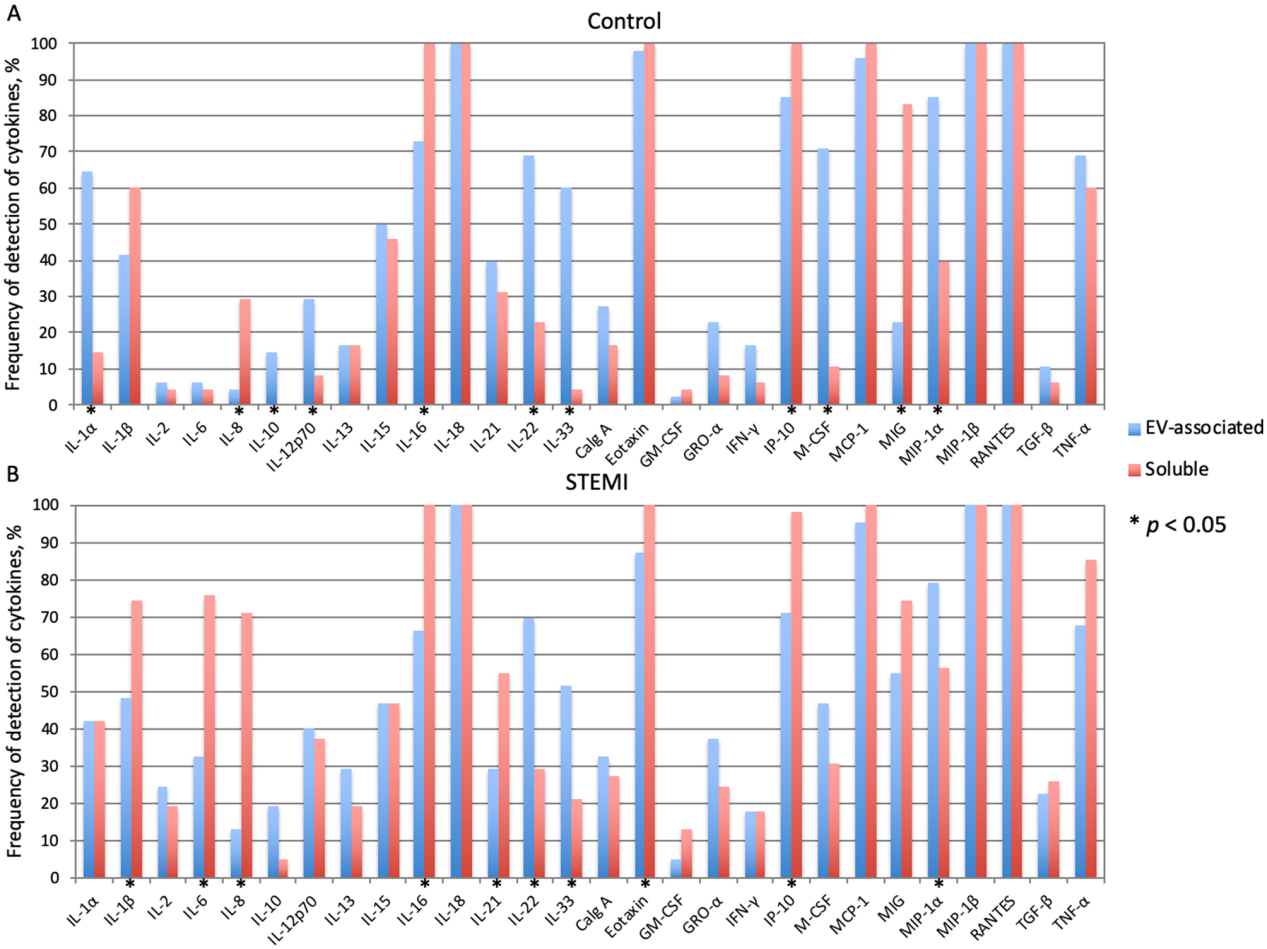
Frequencies of cytokine detection in soluble and EV-associated forms. Data are presented as percentage for all the soluble and EV-associated cytokines in the control group (**A**) and the STEMI group (**B**). Asterix indicates *p*-values < 0.05.

Next, we performed a more thorough analysis of the distribution of cytokines between soluble form and EV-associated form. We found that in control volunteers multiple cytokines and some chemokines (in particular, IL-1α, IL-10, IL-12p70, IL-22, IL-33, M-CSF, and MIP-1α) were detected more often in the EV fraction than in the soluble fraction, while in patients with STEMI only three of them (IL-22, IL-33, and MIP-1α) were detected in EV-associated form more often than in soluble. In contrast, we found a substantial number of chemokines and cytokines in patients with STEMI (in particular, IL-1β, IL-6, IL-8, IL-16, IL-21, Eotaxin, and IP-10) that were detected more frequently in soluble than in EV-associated form, while in controls only three of them (IL-8, IL-16, and IP-10), as well as MIG, were found in soluble form more often than in EV-associated form (Fig. 5, also see Supplementary Fig. S1 online). Furthermore, in controls the majority of cytokines were detected independently either in soluble form or in EVs, while in patients with STEMI half of the measured cytokines were simultaneously detected in the same individuals in both EV-associated and soluble forms (see Supplementary Fig. S2 online).

Thus, patients with STEMI compared with controls are characterized by generally higher concentrations of cytokines as well by the higher frequencies of their detection. Moreover, these cytokines in patients with STEMI were simultaneously released in soluble and EV-associated form with a more frequent detection as soluble substances. At the same time, in controls the EV-associated release of cytokines was more prevalent and independent from the release in soluble form.

### Comparison of cytokine profiles in patients with STEMI and controls

**Figure 6:**
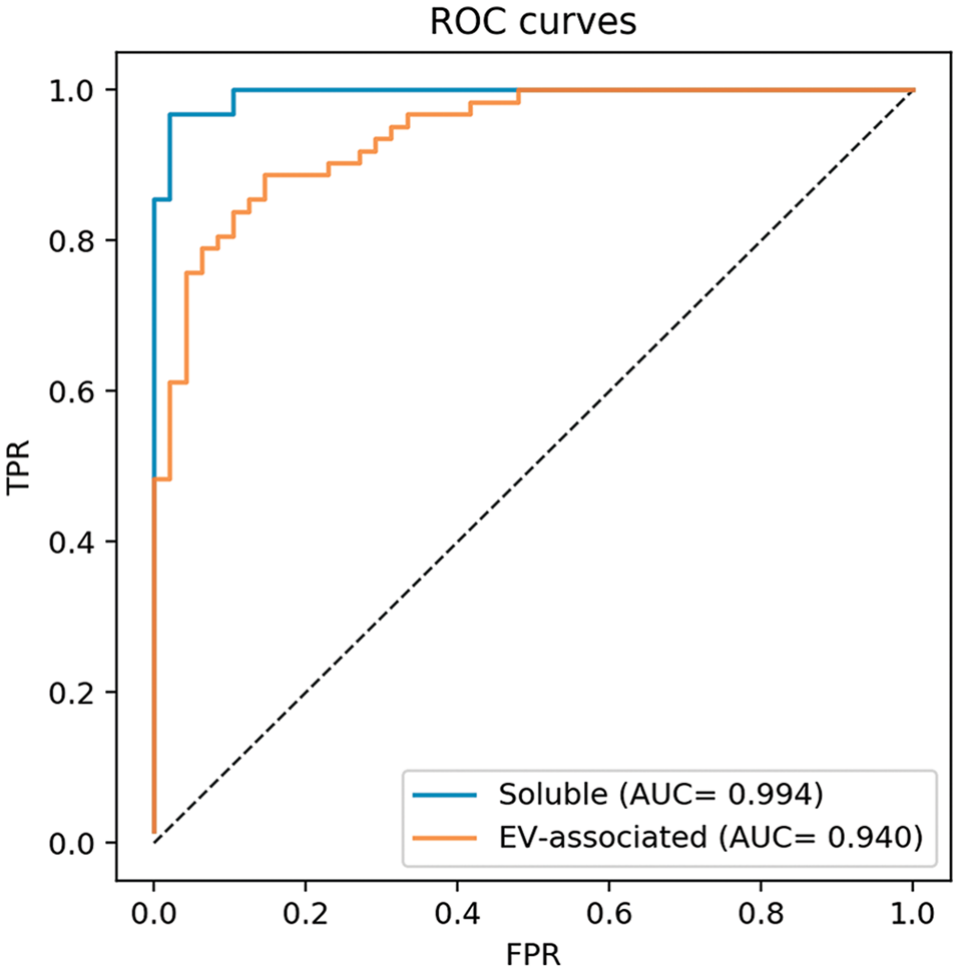
Logistic regression analysis for cytokine concentrations in patients with STEMI and controls. Presented are the ROC-curves for logistic regression models of between-group differences in concentrations of soluble and EV-associated cytokines. TPR, true positive rate; FPR, false positive rate; AUC, area under ROC curve.

To assess the cumulative significance of differences in expression profiles of single cytokines, found between controls and patients with STEMI on the previous step, we used logistic regression analysis. First, we evaluated the models’ mean accuracy, sensitivity, and specificity (see Supplementary Table S2 online). By evaluating either soluble or EV-associated cytokines we found that this model reliably distinguished patients with STEMI from volunteers without CVD with 99.4% and 94.0% areas under the ROC-curves, respectively (Fig. 6). Differentiation between the two groups of individuals was slightly better in the case of soluble cytokines than in EV-associated ones (96.8% vs 83.9% sensitivity and 97.9% vs 89.6% specificity, respectively).

Since our group of patients with STEMI differed from the group of volunteers without CVD in sex, age, and other clinical characteristics, we assessed the effects of these parameters on our regression models. We performed a two-sided Mann–Whitney rank test and found that among all the cytokines only the amounts of soluble IL-6, IL-18, MCP-1, and MIP-1β differed significantly between males and females (see Supplementary Fig. S3 online). However, the significance of differences between males and females (according to *U-* and *p*-values) for these four cytokines was much lower than that between patients with STEMI and controls. Thus, although they were a confounding factor, sex differences did not affect the reliability of the separation of the STEMI group from the controls (see Supplementary Fig. S4A online). Nor did age affect the reliability of this separation, since only soluble MCP-1 and MIP-1β levels were weakly correlated with age (see Supplementary Fig. S5 online) and did not significantly change the regression model (see Supplementary Fig. S4B online). Moreover, we did not find significant confounders between the cardiovascular risk factors (see Supplementary Fig. S6 online).

Thus, by comparison of cytokines in soluble and EV-associated forms it was possible to distinguish with high accuracy between patients with STEMI and controls.

### Clusterization of cytokines by their levels and co-expression in patients with STEMI and controls

**Figure 7:**
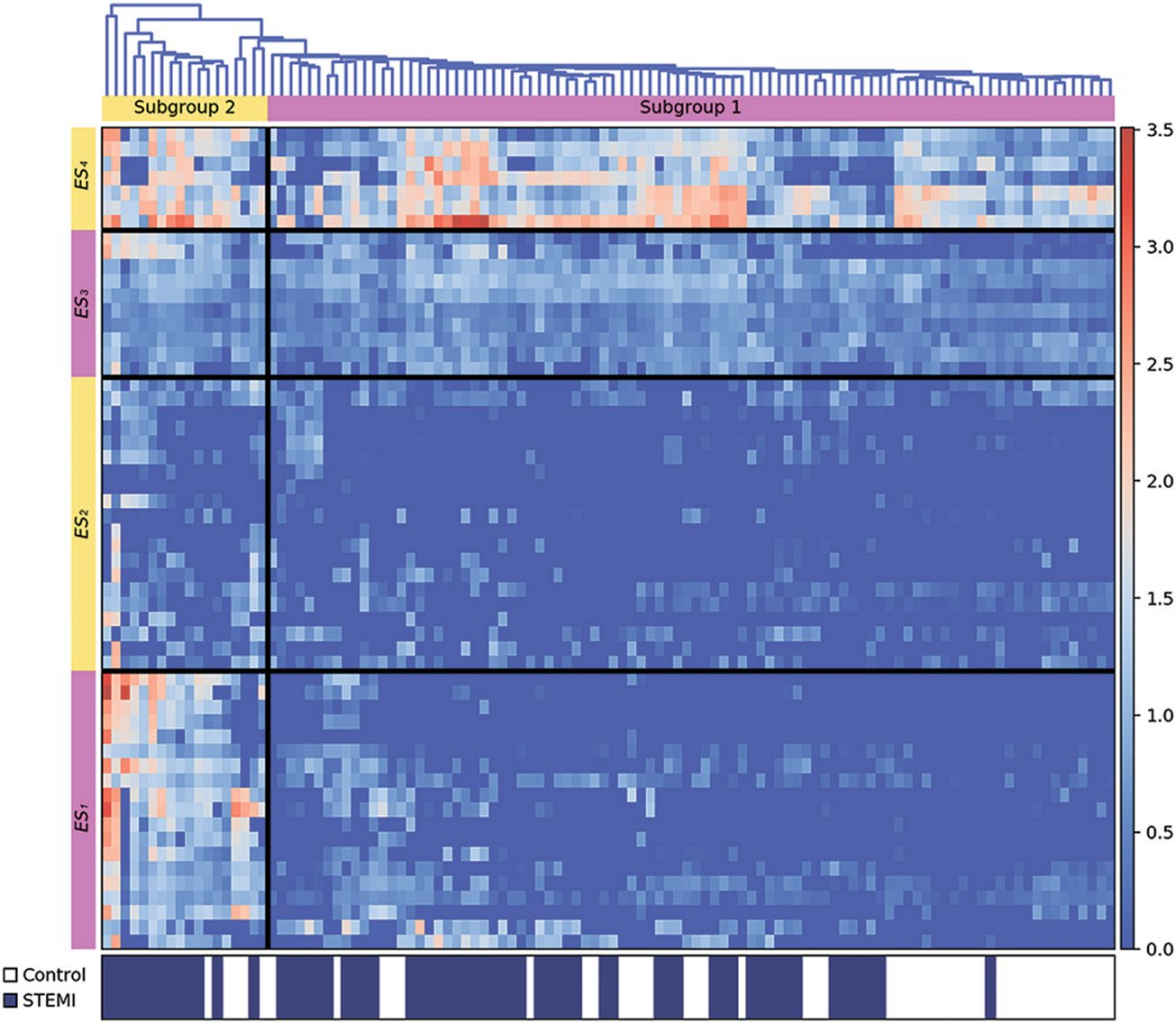
Hierarchical cluster analysis of cytokine concentrations in patients with STEMI and controls. Shown are the log10-normalized values with subtracted mean values for each cytokine of tested individuals. Each row corresponds to one individual, and each column to one cytokine in either soluble or EV-associated form. Red color corresponds to higher expression, and blue to lower. The clustering is presented on the top as a dendrogram. The color legend for the groups is presented at the bottom with the columns marked with white for controls and with blue for patients with STEMI. ES, expression set.

For the further assessment of the differences in the interrelations of studied cytokines in a cumulative model, we performed a cluster analysis with clustering on cytokine fractions and the diagnosis of STEMI together. For the resulting clustering, see Fig. 7.

We found that the distribution of expression of most cytokines (both soluble and EV-associated) in patients with STEMI did not differ from that in controls, whereas the levels of these cytokines in patients with STEMI were in general higher than in controls. At the same time, we identified one set of cytokines that according to the levels of expression was distributed differently between patients with STEMI and controls. This set of cytokines consisted of both soluble and EV-associated forms of IL-1β, IL-12 p70, IL-21, Calg A, Gro-α, IFN-γ, MIP-1α, and TGF-β, as well as of the soluble form of IL-6, and of EV-associated forms of MIG and TNF-α. These cytokines showed high expression in a subgroup consisting of mostly patients with STEMI (Subgroup 2 on the Fig. 7), compared with all the other studied individuals, in whom their levels were close to zero (Subgroup 1 on Fig. 7, also see Supplementary Fig. S7 online). However, the predominance of patients with STEMI in Subgroup 2 did not affect the above-mentioned results of statistical discrimination between two studied groups. We found that even after deletion of this subgroup of patients from the main analysis the differences between groups of patients with STEMI and controls remained largely unchanged (see Supplementary Fig. S8 online).

**Figure 8:**
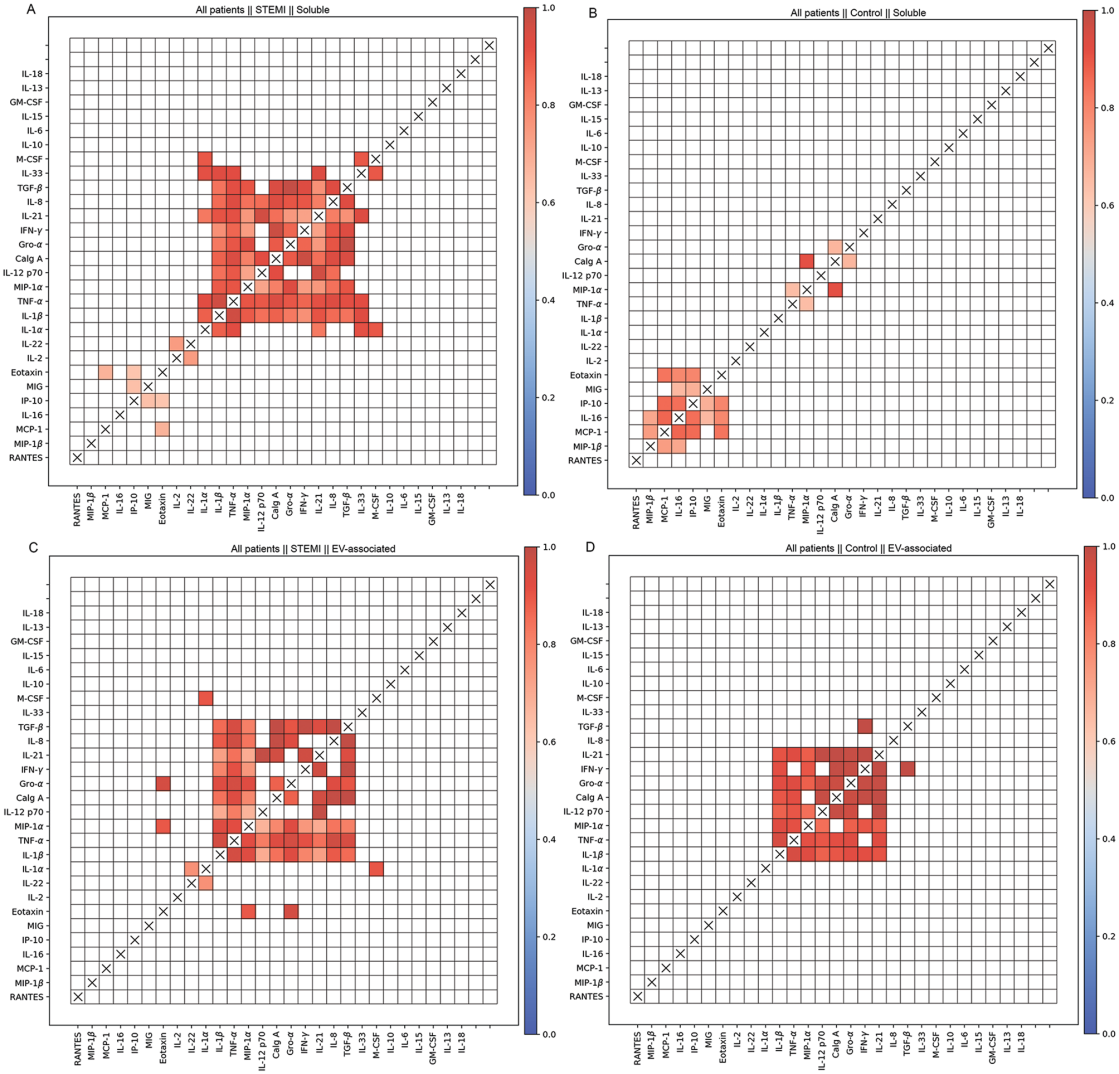
Correlations of cytokine concentrations in all individuals. Shown are significant Pearson R-values for pairwise correlations of different cytokines in all individuals: (**A**) Soluble cytokines in patients with STEMI; (**B**) Soluble cytokines in controls; (**C**) EV-associated cytokines in patients with STEMI; (**D**) EV-associated cytokines in controls. All the correlations are positive.

At the same time, we also performed a cluster analysis of the correlations between the levels of cytokine expression in controls and patients with STEMI for all pairs of cytokines in the soluble and EV-associated forms. All the significant correlations revealed in this analysis were positive. In contrast to the standard cluster analysis, we found that on the basis of their correlations soluble cytokines formed two distinct clusters in control individuals and patients with STEMI (Fig. 8A,B). The strongest positive pairwise correlations for soluble cytokines in patients with STEMI were found between IL-1β, IL-8, IL-12p70, IL-21, Calg A, Gro-α, IFN-γ, MIP-1α, TGF-β, and TNF-α. In controls we only found a small cluster of positive correlations, consisting mostly of chemokines (IL-16, IP-10, MCP-1, MIG, MIP-1β, and Eotaxin). In contrast, the correlations of EV-associated cytokines were clusterized in patients with STEMI in the same fashion as soluble ones and formed a smaller but similar cluster in controls as well (including IL-1β, IL-12p70, IL-21, Calg A, Gro-α, IFN-γ, MIP-1α, and TNF-α) (Fig. 8C and D).

Thus, with a cluster analysis we confirmed our results shown in the monoparametrical analysis. Compared with controls, STEMI was associated with a higher expression of most cytokines (both soluble and EV-associated), with some of them present almost exclusively in patients with STEMI. At the same time cytokines in patients with STEMI were released in a coordinated way, forming a similar cluster in both soluble and EV-associated forms, while in controls the release of EV-associated cytokines was much more coordinated than that of soluble ones, indicating a basic system of intercellular communication.

## Discussion

Dysregulated release of cytokines is widely studied as one of the important complications of several diseases^7^. Also, dysregulated cytokine patterns were shown to be associated with HIV infection^29,30^, CAR T-cell therapy^31^, as well as with pathologies for which etiological agents have not been established, e.g. certain complicated pregnancies^3^ and, in particular, atherosclerosis and acute myocardial infarction that was the focus of our current study^1,2,8–12,32^.

Progression of atherosclerosis is strongly associated with chronic immune activation^33–35^, stimulating the development of anti-inflammatory treatment for patients with ACS^36^. Moreover, the levels of different cytokines also significantly increase in patients during the development of ACS^12,32,37^, especially in those with the most severe form of the disease, STEMI^11,38,39^.

General cytokine profiles in patients with STEMI have been reported previously^11,12,39,40^. The novelty in our analysis compared with published data is the bioinformatics approach to cytokines as a complex redundant network. Indeed, in this framework they act as clusters rather than as individual factors and therefore should be analyzed together^3,41^. Moreover, recently published data indicate that there are at least two systems of cytokines: one consists of soluble molecules and another of cytokines associated with EVs^17–19^. There is a complex relation between these two systems. However, most of the publications on CVD patients have reported on the upregulation of single soluble cytokines, not reflecting the above-described complexity. Here, we analyzed the patterns of expression and clustering of 33 cytokines released as soluble and EV-associated molecules in patients with STEMI and in control individuals.

We found that STEMI was associated with a coordinated upregulation of several cytokines that formed various clusters based on correlations in their modulations and the levels of their expression. This upregulation of cytokines reflects the classical acute inflammatory response to myocardial injury during acute myocardial infarction^11,12^. Also, the abnormal immune activation associated with the destabilization of atherosclerotic plaques may be related to this cytokine upregulation^2,8^. Thus, it has been suggested that the upregulated cytokines may contribute to plaque destabilization^9^, while the complex of IL-1β and IL-18 activated via inflammasome induces expression of adhesion molecules on endothelial cells and macrophages in atherosclerotic plaques, leading to adhesion of new immune cells resulting in plaque destabilization and development of ACS^9,10,32,40,42,43^. Moreover, application of inflammasome inhibitor leads to a decrease of inflammatory response and infarct size in experimental studies^44^, and IL-1β antagonist reduces complications in patients with STEMI^45^. Elevated levels of TNF-α and its receptor were found to increase absorption of lipoproteins in atherosclerotic plaques, leading to a higher complication rate in patients with CVD^10,46,47^. Also, the increased risk of cardiovascular complications was found to be associated with IL-8, a major chemoattractant for monocytes and neutrophils during the acute inflammatory response^12,38,48^. Other chemokines, which attract immune and smooth muscle, not only play a significant role in atherosclerotic plaque development^49^, but also increased infarct size and enhanced myocardial remodelling in clinical studies of acute myocardial injury^50^. In contrast, IL-2 decreased infarct size and myocardial remodelling associated with the expansion of T regulatory cells within the area of myocardial injury^51^.

Other papers, although they did not suggest particular mechanisms, nevertheless linked upregulation of some cytokines and ACS. IL-6 increased the risk of cardiovascular outcomes in patients with STEMI^39^, and its antagonist was used to reduce inflammation through the decrease of C-reactive protein and cardiac troponin T levels in patients with myocardial infarction^52^. IL-12 was also found to increase the risk of cardiovascular events^53^.

Moreover, the upregulation of the cytokines listed above was linked by positive correlations. While clusterization of cytokines in the framework of their network has been found in other systems^19,28^, it has not been reported for CVD. Moreover, we found that correlations between cytokines were different in patients with STEMI in comparison with controls: whereas in the latter group the strongest correlations were predominantly found between chemokines, in patients with STEMI clusters included mostly cytokines. Furthermore, there was a group of cytokines that were virtually absent in controls but were highly expressed in patients with STEMI and were strongly linked to each other with positive correlations between their changes. They included several chemokines that attract T cells, monocytes, and neutrophils during the inflammatory response^49,50^ as well as cytokines that were earlier identified as markers of inflammation associated with cardiovascular outcomes^10–12,32,37,39,42,46,53,54^.

Whether these cytokines cluster because they are released by the same cell or by different cells communicating with each other to release cytokines in a coordinated way remains to be understood. Whichever of these mechanisms is true does not affect the result of our analysis, which not only demonstrated that STEMI is associated with the upregulation of certain groups of cytokines but also showed that some cytokines are released in coordinated fashion. Thus, STEMI affected not only individual cytokines but also the entire cytokine network.

The data discussed above concern soluble cytokines. However, recently a new system of cytokines, those associated with EVs, was discovered^18,19^. EVs are now considered to be universal vehicles that transfer bioactive compounds from one cell to another using “address molecules”^55^. While a majority of the studies demonstrated the importance of EVs as carriers of microRNAs^13–16^, our and others’ recent publications have demonstrated that EVs can carry bioactive proteins, in particular cytokines, and that by delivering these molecules directly to the target cells’ receptors EVs can modify the recipient cell physiology^17,18,28^. As we demonstrated earlier, exogenously added soluble cytokines do not bind to EVs^19^, suggesting that cytokine association with EVs is not an artifact of EV isolation and that this association is not due to nonspecific binding but is rather more likely specific in the course of EV release by the cells.

The release of cytokines within or on the surface of EVs may offer a number of advantages, including stability due to the protection from degradation^19,56^, a longer half-life in circulation, and thus efficient delivery to distant targets^57^. Since cytokines that are encapsulated in EVs are not measured in standard cytokine assays^19^, although several changes in EV-associated cytokines have been reported for several pathologies^17,18,27^, the present work is the first report on EV-associated cytokines in patients with STEMI.

In agreement with our earlier study of soluble and EV-associated cytokines under normal conditions, we found here that these two systems of cytokines behave differently. Some cytokines were released predominantly in EV-associated form while others were released mostly as soluble molecules, indicating the important physiological differences between these groups. Moreover, this difference between EV-associated and soluble cytokines did not correspond to the current cytokine classification.

In contrast to the soluble cytokines that changed significantly in patients with STEMI, EV-associated cytokines were less affected by this disease, except for a few that were upregulated in patients with STEMI in soluble form as well, including IL-2, IL-6, IL-18, and MIG. Furthermore, clusterization of EV-associated cytokines was similar in patients with STEMI and controls. The finding that EV-associated cytokines are less affected by STEMI than soluble ones indicates a basic function of such association between EVs and cytokines in supporting main human physiology. Therefore, the few above-mentioned EV-associated cytokines that were found to be upregulated in patients with STEMI may be particularly important for the CVD-related pathologies.

In particular, IL-2 enhances T regulatory cell expansion within the injured myocardium^51^ and attenuates the development of atherosclerotic plaques^58^. IL-6 is produced mostly by macrophages, vascular smooth muscle cells, and endothelial cells and plays a pivotal role in both atherosclerotic plaque progression and development of complications in STEMI^39,59–61^. Moreover, the level of IL-6 in patients with CVD correlates with the concentration of EVs^62^, and the protection from degradation can explain its preferable packaging into EVs^56^. MIG (CXCL9) is an IFN-inducible chemokine, produced mainly by macrophages and endothelial cells^49^. It induces T cell polarization into Th1/Th17 and facilitates recruitment of activated T cells to the sites of plaque development as well as to the areas of myocardial injury during myocardial infarction^49,63,64^. IL-18, synthesized as an inactive precursor, is highly expressed in macrophages^9^, processed intracellularly via inflammasome activation, and correlates with both atherosclerotic plaque formation and cardiovascular complications^40,43^. At the same time, inflammasome activation was also found to induce secretion of EVs^65,66^ and, in particular, EVs containing IL-1β and IL-18^67–69^.

The range of cytokines associated with EVs differs from that in the soluble form^18,19^ and, therefore, the EV-associated cytokines may have more specific cell targets than the soluble ones^70^. Despite numerous publications, the exact pathways of different EVs remain largely unknown, and its study was outside of the framework of the present work. Future studies may explain functional relations between the increased release of EVs in patients with chronic or acute forms of CVD^20–24,71^, inflammatory activation^72,73^, and the worsening of the outcomes in patients with STEMI^25,26^.

Altogether, the changes in free and EV-associated cytokines and their clusterization allowed the distinction of control plasma from that of patients with STEMI with high accuracy. While it is well established that cytokine secretion during both chronic and acute inflammatory proceses is a hallmark of CVD, in particular of STEMI, a potential etiological agent that could have triggered such immunoactivation was not determined. Several publications indicate that different infections, in particular, activated cytomegalovirus could be such an agent^74–77^. It has now to be established whether the cytokines belonging to the clusters identified in the present work contribute to STEMI and what are the main triggers of their secretion.

## Methods

### Characteristics of patients

A total of 48 control volunteers without CVD (14 men and 34 women, mean age 49.1 ± 9.3 years) and 62 patients with STEMI (52 men and 10 women, mean age 56.7 ± 9.9 years) were enrolled consecutively in our study. Our groups differed significantly in sex and age (*p* < 0.05).

All patients with STEMI were hospitalized in the *A.I. Davydovsky* Moscow City Hospital (Moscow, Russia). Inclusion criteria for patients with STEMI were determined according to the existing guidelines^78^. Exclusion criteria for patients were symptoms of infectious diseases, any record of neoplasms, cardiogenic shock, and thrombolytic therapy. During transportation to the hospital and prior to blood sampling all patients were treated with dual antiplatelet therapy. In hospital, all patients were subjected to standard pharmacological therapy according to the current guidelines^79,80^, including dual antiplatelet therapy, anticoagulants, statins, beta-blockers, and ACE inhibitors.

Control volunteers without CVD were examined in the Diagnostic Centre of the *A.I. Davydovsky* Moscow City Hospital. Absence of CVD was established according to their clinical history, echocardiography, and carotid artery ultrasound. Nevertheless, most of the volunteers have a history of cardiovascular risk factors, such as arterial hypertension, obesity, dyslipidaemia, and smoking. The details of patients’ and volunteers’ characteristics are shown in Supplementary Table S3 online.

The study protocol, the study methods and the form of participants’ consent were approved by the Interuniversity Committee of Ethics of Moscow State University of Medicine and Dentistry and were carried out in accordance with the ICH-GCP rules and the interuniversity guidelines and regulations.

### Blood plasma collection

Blood samples were collected within the first hour of patient admission (before coronary angiography) or during the visit of volunteers without CVD. All participants provided written informed consent prior to the collection of biological samples. The utilization of blood samples for research purposes was approved by the by the Interuniversity Committee of Ethics of Moscow State University of Medicine and Dentistry. We collected peripheral blood by intravenous withdrawal in vacuum tubes with sodium citrate as anticoagulant (Sarstedt, Nuembrecht, Germany, cat#05.1071.001). The first tube with blood was discarded because it contained platelets activated during venepuncture and skin fibroblasts. We prepared platelet-poor plasma less than 1 h after blood collection by double centrifugation at 3000 g for 15 min. Then, plasma was aliquoted to 300 μl, frozen at − 80 °C, and shipped on dry ice to the Section on Intracellular Interactions of the *Eunice Kennedy Shriver* National Institute of Child Health and Human Development, National Institutes of Health (NICHD/NIH/DHHS) (Bethesda, MD, USA) for cytokine measurement.

### Preparation of EV fractions

We defrosted all plasma samples collected as described above and separated EVs from plasma using ExoQuick (System Biosciences, Palo Alto, CA) according to the manufacturer’s protocol, as described previously^19^. Briefly, ExoQuick was added to plasma without addition of thrombin at a ratio of 63 μl of ExoQuick to 250 μl of plasma and refrigerated for 30 min at 4 °C. ExoQuick/sample mixtures were centrifuged at 1500 × *g* for 30 min to pellet EVs. Supernatant was collected for the measurement of EV-free soluble plasma cytokines. Then, the EV pellet was centrifuged again at 1500 × *g* for 5 min, and all traces of fluid were removed. The pellet was resuspended in 1X phosphate-buffered saline (PBS) in the original volume and mixed with Triton X at a final concentration of 1% for measurement of cytokines on lysed EVs. Both fractions were stored at 4 °C, and cytokine measurement was performed within 24 h.

### Cytokine measurement

Cytokine measurements were performed on EV-free supernatants to evaluate soluble cytokines and on lysed EV fractions to measure cytokines both from the surface and from inside EVs^19^. We evaluated cytokines using an in-house multiplexed bead-based assay for measurement of the following 33 cytokines: IL-1α, IL-1β, IL-2, IL-4, IL-6, IL-7, IL-8 (CXCL8), IL-10, IL-12p70, IL-13, IL-15, IL-16, IL-17, IL-18, IL-21, IL-22, IL-33, Calgranulin A (S100A8, Calg A), Eotaxin (CCL11), granulocyte–macrophage colony-stimulating factor (GM-CSF), growth-regulated alpha (GRO-α or CXCL1), interferon-γ (IFN-γ), interferon-γ-induced protein-10 (IP-10 or CXCL10), interferon-inducible T-cell alpha chemoattractant (ITAC or CXCL11), macrophage colony-stimulating factor (M-CSF), monocyte chemoattractant protein-1 (MCP-1 or CCL2), monokine induced by IFN-γ (MIG or CXCL9), macrophage inflammatory protein-1α (MIP-1α or CCL3), MIP-1β (CCL4), MIP-3α (CCL20), regulated on activation normally T-cell expressed and secreted (RANTES or CCL5), transforming growth factor-β (TGF-β), and tumor necrosis factor-α (TNF-α), as described previously with slight modifications^19,28^. All antibody pairs and cytokine standards were purchased from R&D Systems except those for IL-4 (Biolegend) and IL-21 (eBioscience). Magnetic beads (Luminex) with 33 distinct spectral signatures (regions) were coupled to cytokine-specific capture antibodies according to the manufacturer’s recommendations and stored at 4 °C. All cytokine pairs were verified to be free of cross-reactivity. Standards and samples were diluted in assay buffer (1X PBS with 20 mM Tris–HCl, 1% each normal mouse and goat serum (Gemini Bioproducts), and 0.05% Tween 20), combined with bead mixtures, and incubated overnight at 4 °C. Calculations of lysed EV concentrations were based on standard curves performed in lysis buffer. Plates were washed three times and incubated with mixtures of polyclonal biotinylated anti-cytokine antibodies (R&D Systems) in assay buffer for 1 h at room temperature. Plates were washed three times and incubated for 25 min with streptavidin–phycoerythrin (Invitrogen) at 16 μg/ml in PBS. Plates were washed three times and beads were resuspended in PBS. We read plates on a Luminex 200 analyzer with acquisition of a minimum of 100 beads for each region and analyzed them using Bioplex Manager software (BioRad). We determined cytokine concentrations using 5P regression algorithms with different lower limits of detection (LLOD) for each cytokine: IL-1α (51)—1.5 pg/ml, IL-1β (67)—3 pg/ml, IL-2 (37)—0.6 pg/ml, IL-4 (82)—1.2 pg/ml, IL-6 (53)—0.6 pg/ml, IL-7 (88)—0.6 pg/ml, IL-8 (84)—0.6 pg/ml, IL-10 (9)—25 pg/ml, IL-12 (22)—1.2 pg/ml, IL-13 (86)—89 pg/ml, IL-15 (55)—3.5 pg/ml, IL-16 (35)—9 pg/ml, IL-17 (76)—0.9 pg/ml, IL-18 (27)—0.3 pg/ml, IL-21 (20)—97 pg/ml, IL-22 (69)—75 pg/ml, IL-33 (18)—87 pg/ml, Calg A (80)—22 pg/ml, Eotaxin (72)—304 pg/ml, GM-CSF (42)—1.2 pg/ml, Gro-a (12)—7.5 pg/ml, IFN-γ (33)—35 pg/ml, IP-10 (46)—36 pg/ml, ITAC (25)—35 pg/ml, M-CSF (44)—6 pg/ml, MCP-1 (65)—18 pg/ml, MIG (63)—60 pg/ml, MIP-1α (61)—18 pg/ml, MIP-1β (29)—3 pg/ml, MIP-3α (78)—97 pg/ml, RANTES (57)—6 pg/ml, TGF-β (59)—13.8 pg/ml, and TNF-α (48)—2 pg/ml. Although the threshold of detection is based on the characteristics of the assay used and does not represent a clinically significant cutoff value, for the statistical analysis, a cytokine in samples was considered “detected” if its concentration was higher than the LLOD of the assay used, and “not detected” when it falls below the LLOD.

### Statistical analysis

Statistical analysis was performed with Python-2. The expression values obtained in the present study were in most cases not normally distributed, according to the Shapiro–Wilk test, and are represented as medians and interquartile ranges (Q1–Q3). Since distributions were not normal, for comparison of two groups we used the Mann–Whitney rank test with continuity correction. For the analysis of categorical parameters, we used a two-tailed Fisher’s exact test with 2 × 2 frequency tables. In order to overcome errors from multiple comparisons we performed a Benjamini–Hochberg FDR-correction with calculation of critical values for each comparison matched with corresponding *p*-values; we calculated adjusted *p*-values and compared them with a critical value of 0.05; below, ‘*p*-value’ refers to Benjamini–Hochberg adjusted *p*-values, if not stated otherwise. For a heatmap data visualisation *p*-values are shown as log_10_ from original *p*-values, where log_10_ *p*-values ≤ − 1.3 correspond to *p*-values < 0.05. For the age distribution we made the assumption of its normality and analyzed this distribution using the *t*-test.

To determine the significance of differences between patient and control groups, logistic regression analysis was performed independently for soluble and EV-associated fractions of cytokines. We calculated the decimal logarithm of the cytokine expression values, then calculated *z*-scores independently for each cytokine and used the resulting values as input data to build a logistic regression classifier. Also, we performed a control for overfitting and stability of regression models. Thereto we randomly split the dataset into training and testing subsets with 3:1 size ratios and used the training subsets to build a logistic regression, estimating the mean accuracy on the resulting models. The whole procedure was repeated for *n* = 1000 random train/test partitions. This analysis proved the stability of coefficients and the predictive quality of the initial models (see Supplementary Fig. S9, S10, and S11 online).

For the analysis of correlations between different cytokines and between cytokines and patients’ ages, Pearson’s coefficient of correlation and corresponding *p*-value with Bonferroni correction for multiple comparisons were calculated. We performed the same analysis using Spearman correlation coefficients with similar results. Values of corrected *p* < 0.05 were considered statistically significant; graphs are presented with *p*-values in log10-scale. For the assessment of the between-group differences in cytokines in a cumulative model, we performed a hierarchical cluster analysis using log10-normalized values with subtracted mean values for each cytokine. In each case, the UPGMC algorithm with Euclidean metric was used. For the assessment of the correlations between the levels of cytokine expression in cluster analysis we estimated Pearson correlation coefficients *R* and corresponding *p*-values for all pairs of cytokines. Because of the non-normal distribution of cytokines and a number of outliers, in order to estimate the confidence interval (CI) upper and lower values, bootstrapping was performed. We used 2.5 and 97.5 percentile values as 95%-CI boundaries (CI 2.5 and CI 97.5, respectively). Correlations with |R|≥ 0.5 and *p*-values with Bonferroni correction ≤ 0.05 and (CI 2.5 ≥ 0.45 or CI 97.5 ≤ − 0.45) were treated as significant.

## Supplementary information

Supplementary information.
