# Supplementary material for: Differential clusterization of soluble and extracellular vesicle-associated cytokines in myocardial infarction
Source: Sci Rep. 2020 Dec 3;10:21114. doi: 10.1038/s41598-020-78004-y (PMC7713058; doi:10.1038/s41598-020-78004-y)

Differential clusterization of soluble and extracellular vesicle-associated cytokines in myocardial infarction

Anna Lebedeva[1,4], Wendy Fitzgerald[2], Ivan Molodtsov[3], Alexander Shpektor[1], Elena Vasilieva[1*], and Leonid Margolis[2]

[1] Laboratory of Atherothrombosis, Moscow State University of Medicine and Dentistry, 11/6 Yauzskaya Street, Moscow, Russia, 119027

[2] Section on Intercellular Interactions, Eunice Kennedy Shriver National Institute of Child Health and Human Development, National Institutes of Health, Bethesda, Maryland, USA, 20892

[3] N.F. Gamaleya Federal National Research Centre for Epidemiology and Microbiology, 18 Gamaleya Street, Moscow, Russia, 123098

[4] Current affiliation – Charité University of Medicine Berlin, Department of Internal Medicine and Cardiology, Augustenburger Platz 1, Berlin, Germany, 13353

SUPPLEMENTARY MATERIAL

Supplementary Table S1. Cytokine concentrations and frequencies of detection in patients with STEMI and controls without cardiovascular diseases.

Cytokine/Group		Controls (N= 48)			Patients with STEMI (N=62)		
		Median	Q1-Q3	%	Median	Q1-Q3	%
Soluble	IL-1α	1.5	§	14.6	1.5	1.5-3.7	41.9
	IL-1β	5.0	3.0-11.3	60.4	8.3	3.2-16.2	74.2
	IL-2	0.6	§	4.2	0.6	§	19.4
	IL-6	0.6	§	4.2	3.4	0.8-6.4	75.8
	IL-8	0.6	0.6-0.9	29.2	3.3	0.6-11.6	71.0
	IL-10	25.0	§	0	25.0	§	4.8
	IL-12p70	1.2	§	8.3	1.2	1.2-3.6	37.1
	IL-13	89.0	§	16.7	89.0	§	19.4
	IL-15	3.5	3.5-6.7	45.8	3.5	3.5-13.0	46.8
	IL-16	71.6	53.3-153.8	100	156.1	100.5-211.4	100
	IL-18	247.5	201.6-353.9	100	457.1	319.2-632.6	100
	IL-21	97.0	97.0-186.7	31.2	168.5	97.0-900.7	54.8
	IL-22	75.0	§	22.9	75.0	75.0-104.4	29.0
	IL-33	87.0	§	4.2	87.0	§	21.0
	Calg A	22.0	§	16.7	22.0	22.0-52.2	27.4
	Eotaxin	9693.6	5865.3-32073.4	100	22321.7	8422.8-54241.5	100.0
	GM-CSF	1.2	§	4.2	1.2	§	12.9
	GRO-α	7.5	§	8.3	7.5	§	24.2
	IFN-γ	35.0	§	6.2	35.0	§	17.7
	IP-10	1370.7	759.3-5465.9	100	3992.4	1161.9-9178.9	98.4
	M-CSF	6.0	§	10.4	6.0	6.0-8.3	30.6
	MCP-1	250.6	162.7-450.7	100	571.1	371.4-872.0	100.0
	MIG	240.5	98.5-1574.0	83.3	1754.2	62.1-5492.6	74.2
	MIP-1α	18.0	18.0-28.4	39.6	22.2	18.0-63.5	56.5
	MIP-1β	60.2	46.8-78.1	100	95.6	72.0-133.1	100
	RANTES	2133.9	1177.8-6806.9	100	2376.1	856.4-6488.0	100
	TGF-β	13.8	§	6.2	13.8	13.8-20.6	25.8
	TNF-α	3.4	2.0-7.7	60.4	9.9	5.0-18.3	85.5
EV-associated	IL-1α	1.9	1.5-3.3	64.6	1.5	1.5-2.8	41.9
	IL-1β	3.0	3.0-6.5	41.7	3.0	3.0-12.2	48.4
	IL-2	0.6	§	6.2	0.6	§	24.2
	IL-6	0.6	§	6.2	0.6	0.6-1.1	32.3
	IL-8	0.6	§	4.2	0.6	§	12.9
	IL-10	25.0	§	14.6	25.0	§	19.4
	IL-12p70	1.2	1.2-1.6	29.2	1.2	1.2-4.3	40.3
	IL-13	89.0	§	16.7	89.0	89.0-153.4	29.0
	IL-15	4.1	3.5-13.0	50.0	3.5	3.5-12.2	46.8

	IL-16	22.2	9.0-35.4	72.9	37.2	9.0-67.6	66.1
	IL-18	10.2	8.5-15.7	100	13.1	10.4-19.0	100
	IL-21	97.0	97.0-291.7	39.6	97.0	97.0-342.8	29.0
	IL-22	169.1	75.0-309.3	68.8	198.8	75.0-292.4	69.4
	IL-33	197.1	87.0-363.9	60.4	114.5	87.0-287.2	51.6
	Calg A	22.0	22.0-45.9	27.1	22.0	22.0-153.2	32.3
	Eotaxin	4264.4	1662.5-6893.5	97.9	2662.7	729.5-9654.8	87.1
	GM-CSF	1.2	§	2.1	1.2	§	4.8
	GRO-α	7.5	§	22.9	7.5	7.5-29.9	37.1
	IFN-γ	35.0	§	16.7	35.0	§	17.7
	IP-10	605.5	197.7-887.4	85.4	520.4	36.0-1591.3	71.0
	M-CSF	10.4	6.0-17.8	70.8	6.0	6.0-14.2	46.8
	MCP-1	77.5	62.3-101.6	95.8	87.3	56.5-115.1	95.2
	MIG	60.0	§	22.9	210.1	60.0-806.6	54.8
	MIP-1α	35.8	27.3-50.1	85.4	40.5	24.7-82.8	79.0
	MIP-1β	25.2	21.4-30.2	100	27.4	20.2-36.0	100
	RANTES	2798.4	940.7-4429.8	100	761.4	406.3-2437.9	100
	TGF-β	13.8	§	10.4	13.8	§	22.6
	TNF-α	3.9	2.0-7.1	68.8	5.5	2.0-13.6	67.7

§ - not enough data for the interquartile range (< 25 % of positive samples).

Supplementary Table S2. Logistic regression analysis of cytokine levels in patients with STEMI and controls without cardiovascular diseases.

	**Soluble**	**EV-associated**
**Mean Accuracy**	0.973	0.864
**Sensitivity**	0.968	0.839
**Specificity**	0.979	0.896

Supplementary Table S3. Clinical characteristics of patients with STEMI and controls without cardiovascular diseases.

	**Patients with STEMI**	**Controls without CVD**	***p*-value**
N	62	48	
Age (years), mean ± SD	56.7 ± 9.9	49.1 ± 9.3	0,0001*
Men, N (%)	52 (83.9)	14 (29.2)	0,0000*
Smoking, N (%)	42 (67.7)	11 (23.9)	0,0000*
Hypertension, N (%)	41 (66.1)	12 (25.0)	0,0000*
Dyslipidemia, N (%)	31 (50.0)	7 (14.6)	0,0001*
Obesity, N (%)	22 (35.5)	18 (37.5)	0.8274

Data are presented as mean ± SD or as number (percentage) of patients.

* Differences are statistically significant at $p < 0.05$.

Supplementary Figure S1. Comparison of frequencies of cytokine detection in soluble and EV-associated forms. Shown is a heatmap of *p*-values for Fisher exact test of comparison of the frequencies of cytokine detection between soluble and EV-associated forms calculated separately for the STEMI group, for the control group, and for all individuals together; *p*-values are presented in log10-scale with Benjamini-Hochberg correction. Log_{10} *p*-values ≤ -1.3 correspond to *p*-values < 0.05.

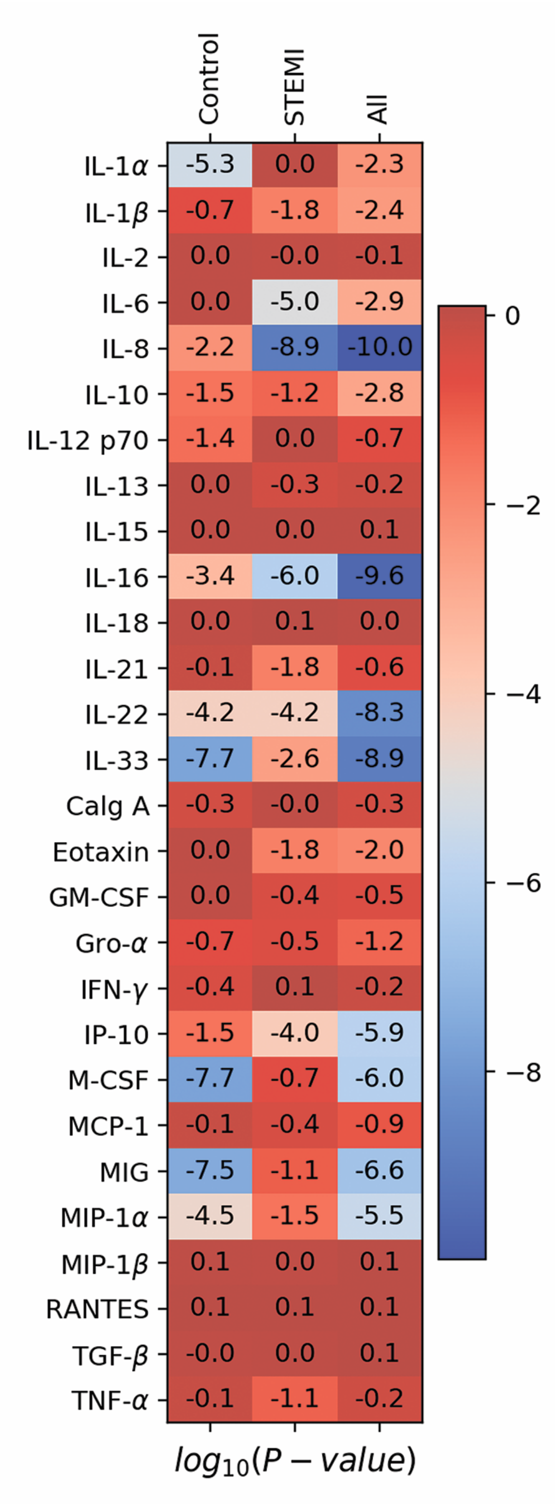

Supplementary Figure S2. Association of frequencies of cytokine detection between soluble form and EV-associated form. Shown is a heatmap of *p*-values for Fisher exact test of dependency of cytokine detection in soluble and EV-associated forms calculated separately for the STEMI group, for the control group, and for all individuals together; *p*-values are presented in log10-scale with Benjamini-Hochberg correction. Log_{10} *p*-values ≤ -1.3 correspond to *p*-values < 0.05.

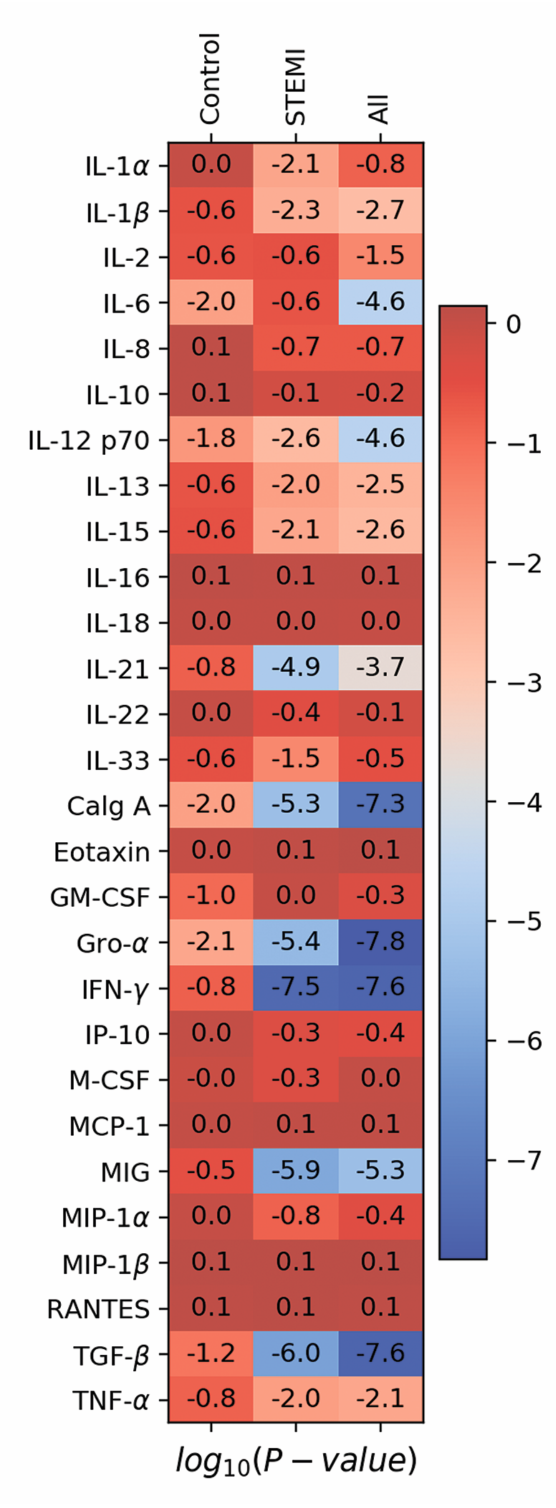

Supplementary Figure S3. Comparison of cytokine levels in males and females for all individuals.

Shown is a heatmap of *p*-values for the Mann-Whitney U-test for comparisons of the levels of soluble and of EV-associated cytokines between males and females; *p*-values are presented in log10-scale with Benjamini-Hochberg correction. Log_{10} *p*-values ≤ -1.3 correspond to *p*-values < 0.05.

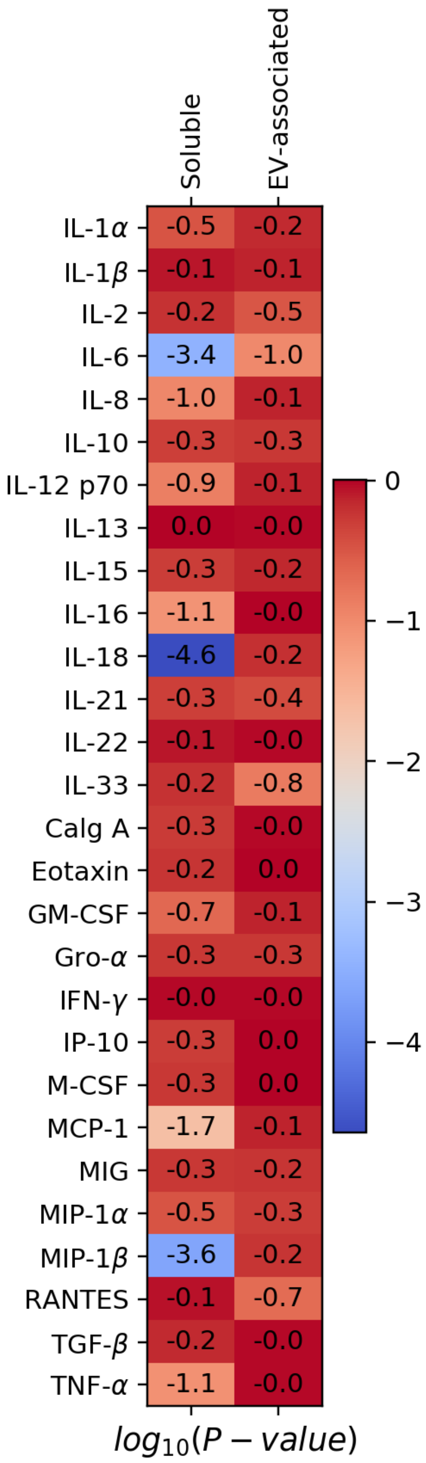

Supplementary Figure S4. Logistic regression analysis for cytokine levels with age and sex as confounding factors in patients with STEMI and controls without cardiovascular diseases.

Shown are the logistic regression coefficients for concentrations of soluble and EV-associated cytokines in the models of between-group differences. A, sex included as confounding factor; B, age included as confounding factor.
The less-than-20% overall changes of the regression coefficients from the original model should not be considered as a confounding factor.

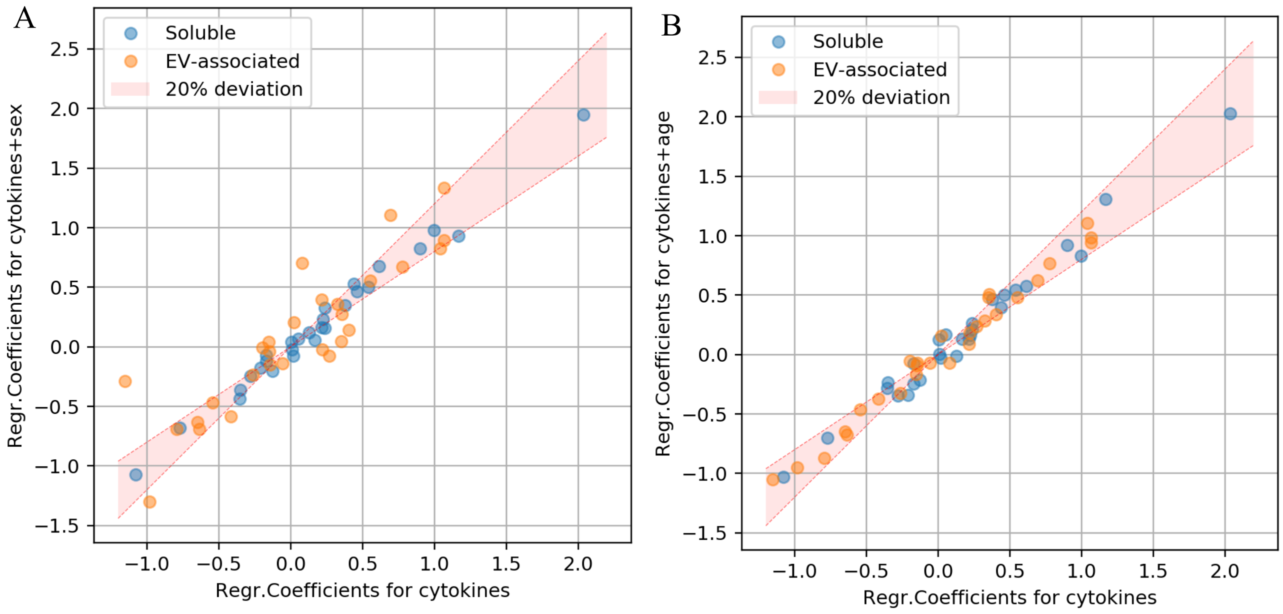

Supplementary Figure S5. Correlations between cytokine levels and age of patients with STEMI and controls without cardiovascular diseases.

Shown are Pearson *p*-values and R-values for correlations of the levels of soluble and EV-associated cytokines and age of tested individuals (patients and controls).

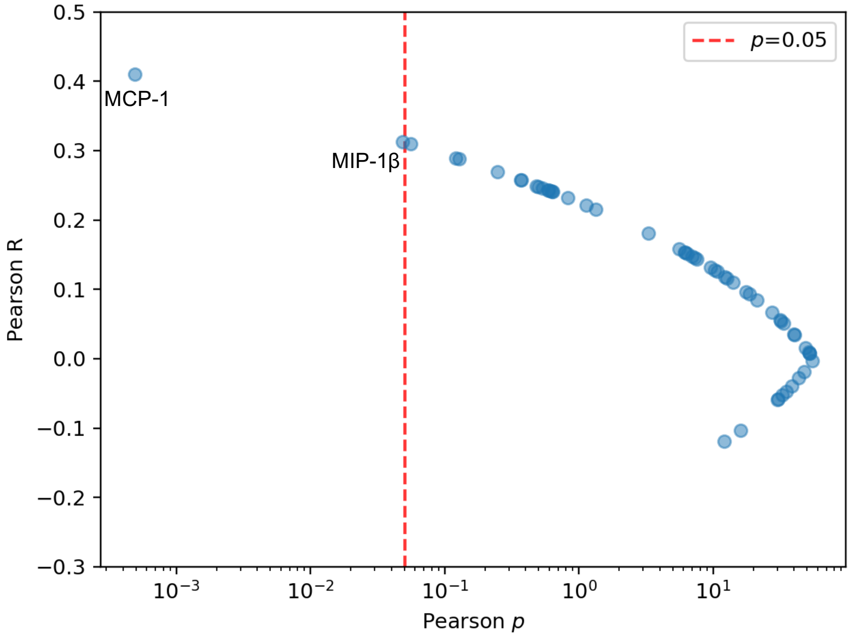

Supplementary Figure S6. Logistic regression analysis for cytokine levels with cardiovascular risk factors in patients with STEMI and controls without cardiovascular diseases.

Shown are the logistic regression coefficients for concentrations of soluble and EV-associated cytokines in the models of between-group differences. A, smoking included as confounding factor; B, obesity included as confounding factor; C, dyslipidemia included as confounding factor; D, hypertension included as confounding factor.
The overall changes of the regression coefficients from the original model were less than 20% and therefore should not be considered as a confounding factor.

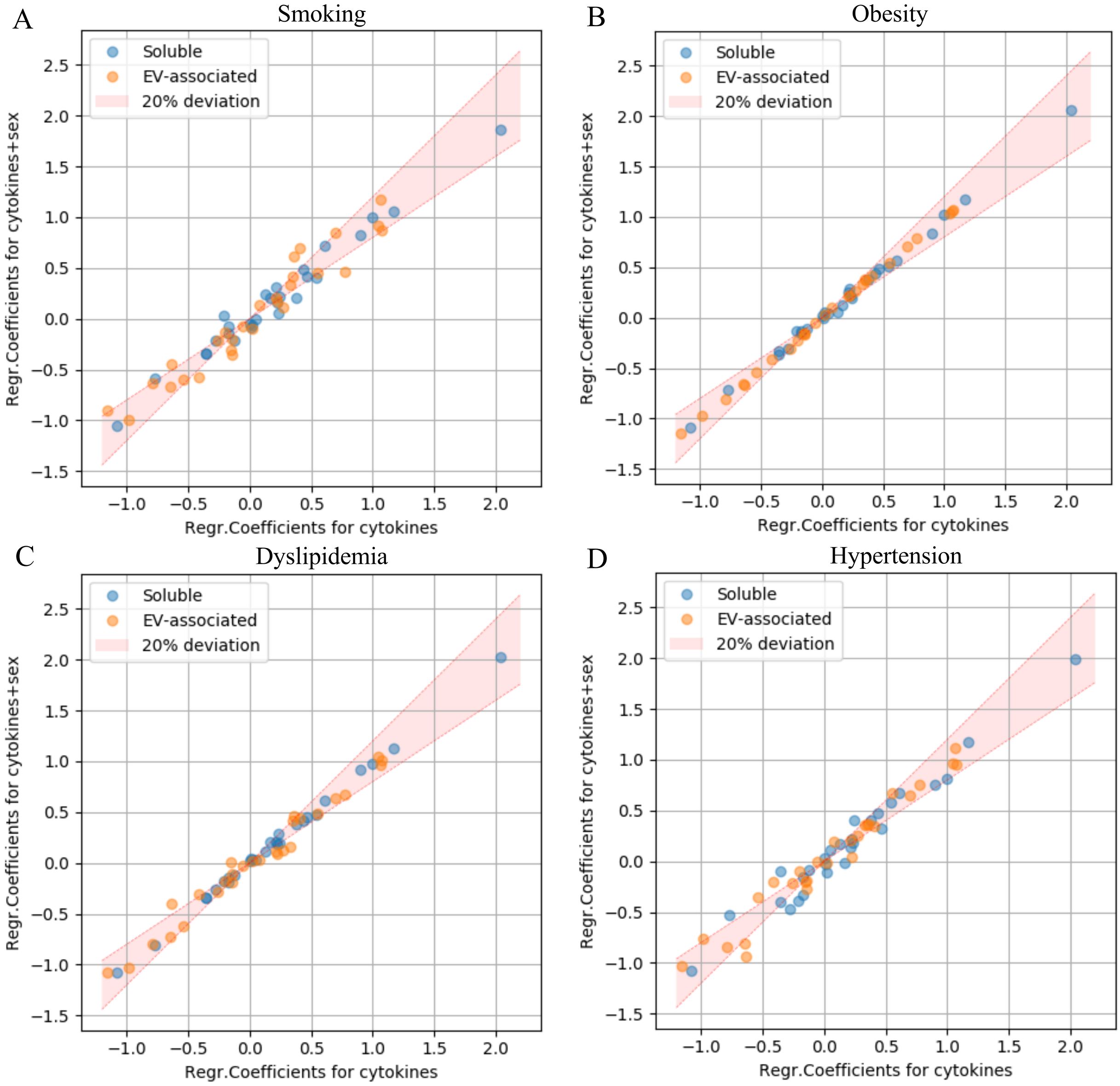

Supplementary Figure S7. Comparison of cytokine levels between subgroup 1 and subgroup 2.

Shown is a heatmap of *p*-values for the Mann-Whitney U-test for comparison of the levels of soluble and of EV-associated cytokines between subgroup 1 and subgroup 2 of all tested individuals; *p*-values are presented in log10-scale with Benjamini-Hochberg correction. Log_{10} *p*-values ≤ -1.3 correspond to *p*-values < 0.05.

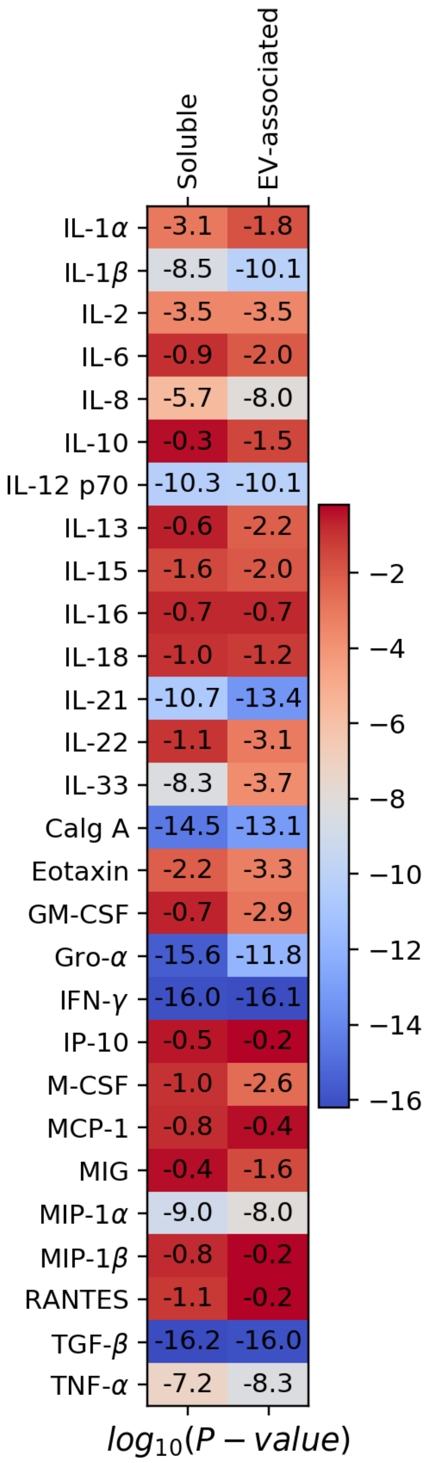

Supplementary Figure S8. Comparison of cytokine levels between STEMI patients and controls in subgroup 1.

Shown is a heatmap of p-values for Mann-Whitney U-test for comparison of the amounts of soluble and of EV-associated cytokines between STEMI patients and controls in subgroup 1; p-values are presented in log10-scale with Benjamini-Hochberg correction. $\log_{10}$ p-values ≤ -1.3 correspond to p-values < 0.05.

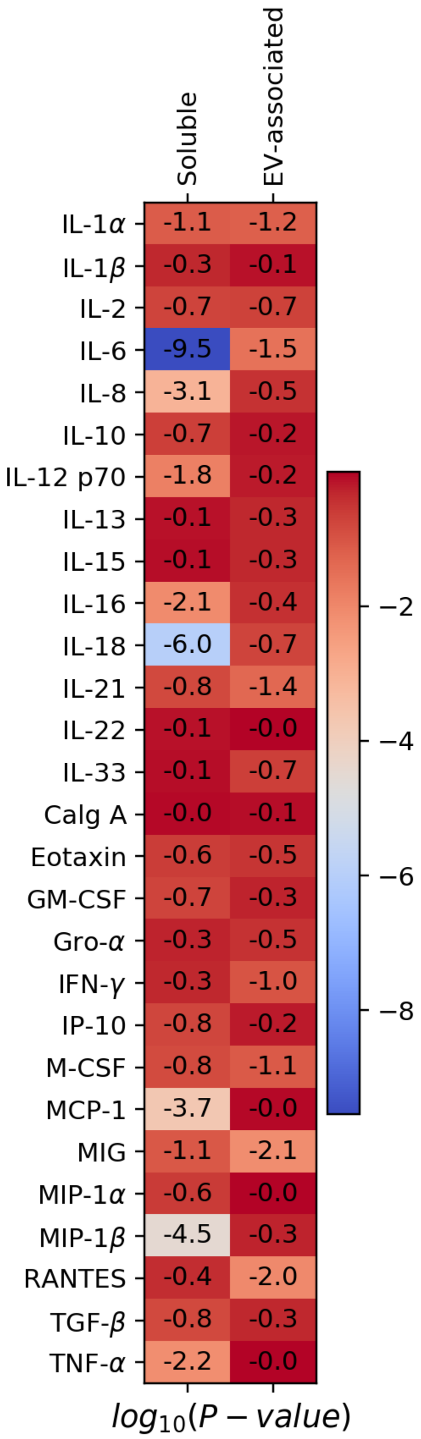

Supplementary Figure S9. Control for overfitting and stability of regression model with soluble cytokines.

Shown are the regression coefficients for all the cytokines in soluble form. The black curve stands for coefficient values from the total model, the green for coefficient values averaged over all partitions, and the red for one standard deviation of confidence interval based on all partitions. Cytokines are sorted by the averaged coefficients.

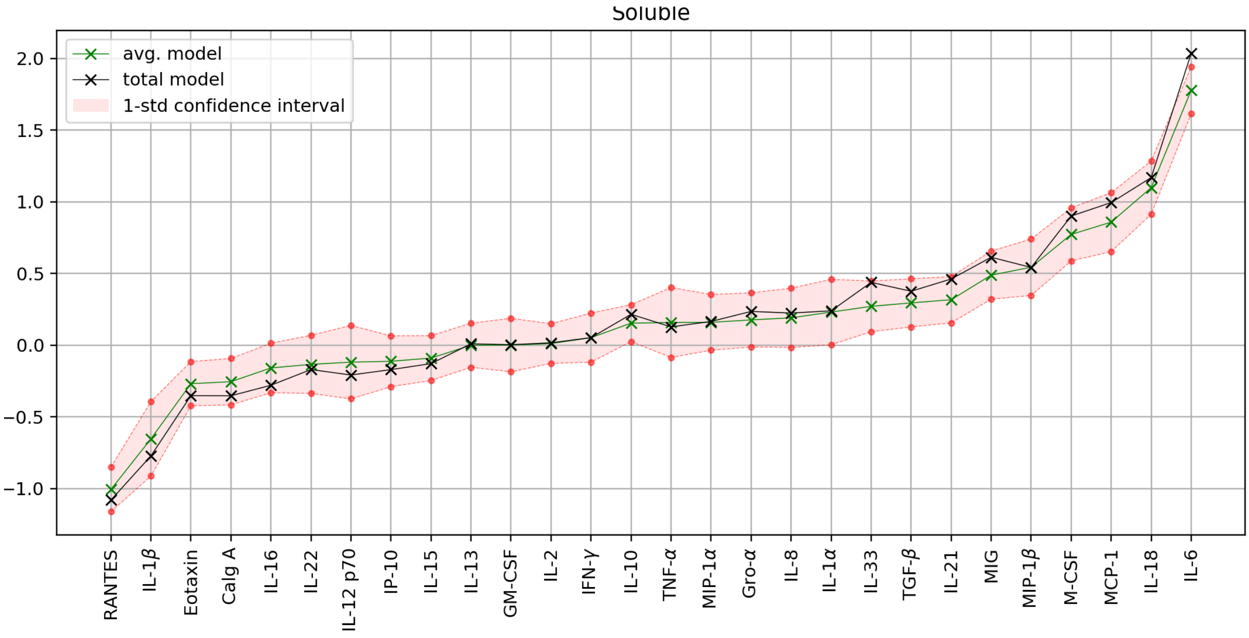

Supplementary Figure S10. Control for overfitting and stability of regression model with EV-associated cytokines.

Shown are the regression coefficients for all the cytokines in EV-associated form. The black curve stands for coefficient values from the total model, the green for coefficient values averaged over all partitions, and the red for one standard deviation of confidence interval based on all partitions. Cytokines are sorted by the averaged coefficients.

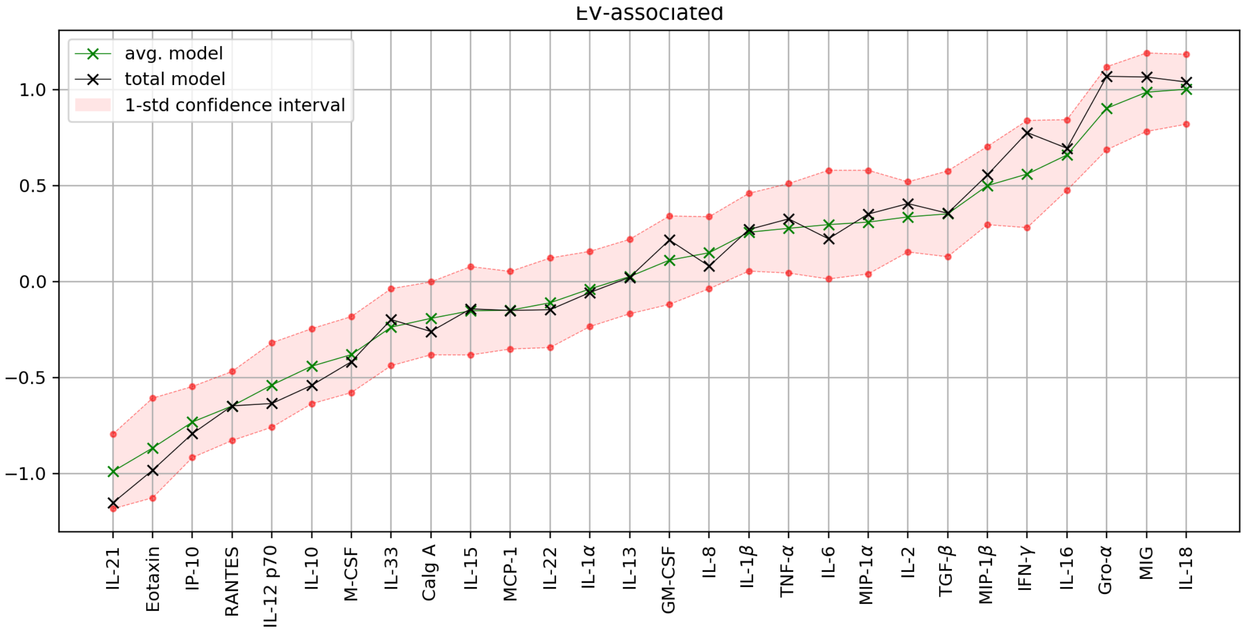

Supplementary Figure S11. Logistic regression analysis for cytokine levels in patients with STEMI and controls without cardiovascular diseases.

Presented are the distributions of mean accuracies for random training/testing partitions for the logistic regression models of between-group differences in concentrations of soluble and EV-associated cytokines.

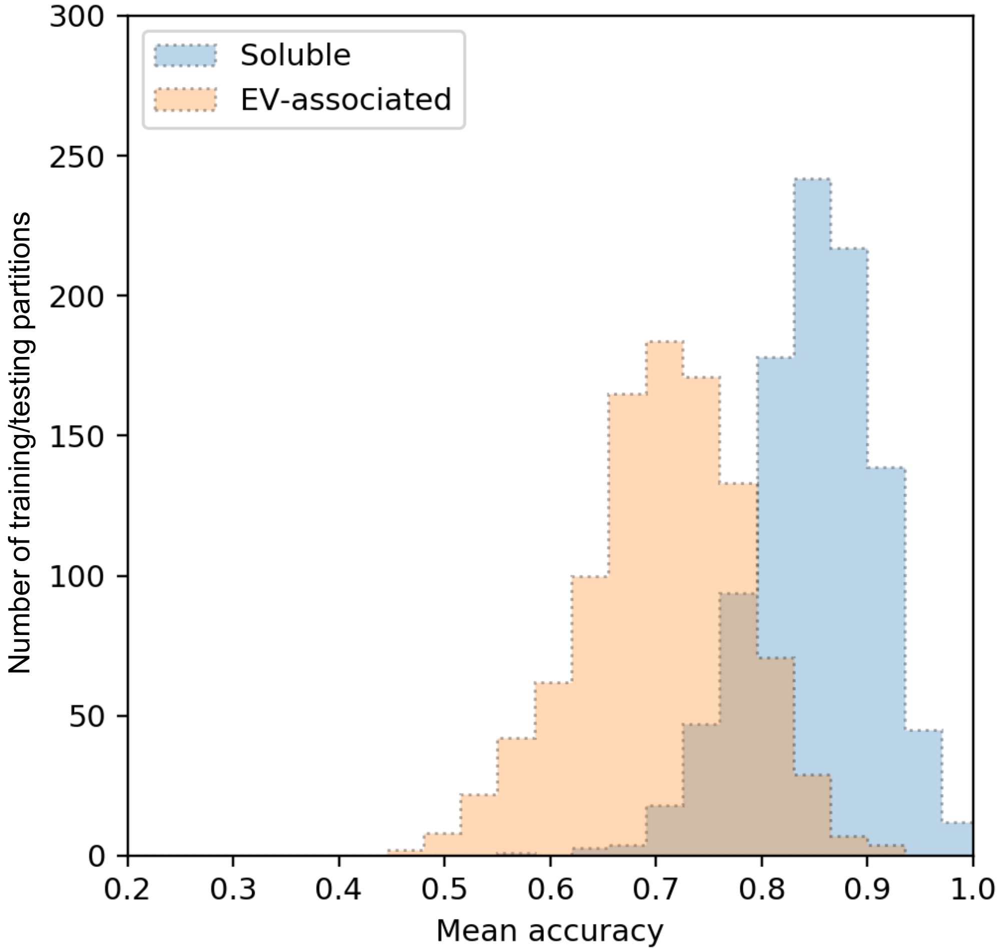

Supplement: Supplementary file 1 — Supplementary information. [file 41598_2020_78004_MOESM1_ESM.pdf]
